# Genome-Wide Discovery of Putative sRNAs in *Paracoccus denitrificans* Expressed under Nitrous Oxide Emitting Conditions

**DOI:** 10.3389/fmicb.2016.01806

**Published:** 2016-11-14

**Authors:** Hannah Gaimster, Lisa Chalklen, Mark Alston, John T. Munnoch, David J. Richardson, Andrew J. Gates, Gary Rowley

**Affiliations:** ^1^School of Biological Sciences, University of East AngliaNorwich, UK; ^2^Earlham Institute (formerly The Genome Analysis Centre)Norwich, UK

**Keywords:** sRNA, regulation, denitrification, soil, NosZ, nitrous oxide

## Abstract

Nitrous oxide (N_2_O) is a stable, ozone depleting greenhouse gas. Emissions of N_2_O into the atmosphere continue to rise, primarily due to the use of nitrogen-containing fertilizers by soil denitrifying microbes. It is clear more effective mitigation strategies are required to reduce emissions. One way to help develop future mitigation strategies is to address the currently poor understanding of transcriptional regulation of the enzymes used to produce and consume N_2_O. With this ultimate aim in mind we performed RNA-seq on a model soil denitrifier, *Paracoccus denitrificans*, cultured anaerobically under high N_2_O and low N_2_O emitting conditions, and aerobically under zero N_2_O emitting conditions to identify small RNAs (sRNAs) with potential regulatory functions transcribed under these conditions. sRNAs are short (∼40–500 nucleotides) non-coding RNAs that regulate a wide range of activities in many bacteria. Hundred and sixty seven sRNAs were identified throughout the *P. denitrificans* genome which are either present in intergenic regions or located antisense to ORFs. Furthermore, many of these sRNAs are differentially expressed under high N_2_O and low N_2_O emitting conditions respectively, suggesting they may play a role in production or reduction of N_2_O. Expression of 16 of these sRNAs have been confirmed by RT-PCR. Ninety percent of the sRNAs are predicted to form secondary structures. Predicted targets include transporters and a number of transcriptional regulators. A number of sRNAs were conserved in other members of the α-proteobacteria. Better understanding of the sRNA factors which contribute to expression of the machinery required to reduce N_2_O will, in turn, help to inform strategies for mitigation of N_2_O emissions.

## Introduction

Nitrous oxide is a potent greenhouse gas with an approximate 300 fold greater radiative potential per molecule than carbon dioxide. In addition to this, it has been described as the biggest single cause of depletion of ozone over the Arctic (Ravishankara et al., 2009). Emissions of N_2_O are continuing to increase every year by approximately 0.25% and once released into the atmosphere it remains stable for around 150 years. The major source (around 70%) of this atmospheric loading of N_2_O is from agriculture, mainly from the use of nitrogen-containing fertilizers by soil microbes. Collectively, these features make N_2_O an important target for mitigation strategies (Richardson et al., 2009).

N_2_O is produced as an intermediate in the sequential reduction of nitrate (${\mathrm{NO}}_{3}^{–}$) to di-nitrogen (N_2_), via nitrite (${\mathrm{NO}}_{2}^{–}$), nitric oxide (NO), and N_2_O, in a process known as denitrification (Zumft and Kroneck, 2006). Reduction of N_2_O to N_2_ by soil microbes is the major biological route for removal of N_2_O (Pomowski et al., 2011). This reaction is catalyzed by a N_2_O reductase, NosZ. However, the increasing emission of N_2_O implies that NosZ is not always able to carry out this removal step in balance with the earlier steps in the denitrification pathway that form N_2_O. It follows that any transcriptional regulation that represses the expression of *nosZ* will in turn reduce the degradation of N_2_O and lead to net emission. Despite the pivotal importance in N_2_O mitigation, transcriptional regulation of NosZ, and other key enzymes involved in denitrification is poorly understood.

*Paracoccus denitrificans* is a soil dwelling member of the α-proteobacteria and is well-studied as a biochemical model for denitrification. The *P. denitrificans* genome encodes biochemical apparatus to switch between aerobic and anaerobic respiration and to utilize a range of electron donors in a modular respiratory network. The genome *P. denitrificans* was sequenced in 2006 and utilizing this, work by our laboratory began to unpick the transcriptome of *P. denitrificans*, cultured under a range of environmentally relevant conditions by using microarrays. This work revealed that the *nos* genes, and therefore N_2_O reduction, are strongly regulated by copper availability (Sullivan et al., 2013). A recent estimate suggested that 20% of Europe’s arable lands are biologically copper deficient (Alloway, 2008). In the presence of limited copper, only basal levels of *nosZ* expression are observed, whereas optimal copper concentrations lead to much higher levels of expression of *nosZ*. This results in transient accumulation of N_2_O in *P. denitrificans* grown in a limited copper media, whereas *P. denitrificans* grown in an optimal copper media do not accumulate N_2_O. This work therefore highlighted an abiotic factor, copper availability, in inducing global changes in gene expression regarding N_2_O emissions (Sullivan et al., 2013). Using the conditions described in this study, we sought to identify and understand if any sRNAs were transcribed and therefore could be playing a role in this key process.

Bacterial sRNAs are an emerging class of regulatory RNAs which are ∼40–500 nucleotides in length. These molecules are found in numerous species of bacteria and until relatively recently, sRNAs were largely an unknown and unexplored area of research. Work by various groups has shown sRNAs can modulate numerous physiological mechanisms and pathways, reviewed in Storz et al. (2011). sRNAs can target either proteins or mRNA transcripts. If the target of a sRNA is a protein, these sRNAs can be further categorized into two distinct groups, the *trans*-acting, and *cis*-acting sRNAs (Gottesman and Storz, 2011). *Trans*-acting sRNAs are defined as those encoded within the intergenic regions of a bacterial genome and act on target RNAs located in distinct locations across the rest of the genome. *Trans*-acting sRNAs can have less than perfect complementarity to their targets and as a result sometimes require a RNA chaperone, Hfq, to facilitate nucleotide binding (Moll et al., 2003). Conversely, *cis*-acting sRNAs originate from the antisense strand of an ORF and sometimes have direct regulatory influence on that particular ORF, though this is not true for all *cis*-acting sRNAs. The recent introduction of RNA-Seq technologies and associated bioinformatic tools has now made the analysis of bacterial transcriptomic data considerably more extensive and efficient.

In this work a combination of RNA-seq alongside bioinformatic approaches were used to gain an insight into the sRNA landscape of *P. denitrificans* was cultured anaerobically under high N_2_O and low N_2_O emitting conditions, and aerobically under zero N_2_O emitting conditions. The aim of this study is to understand the global sRNA profile in *P. denitrificans* as sRNAs could potentially be a valid target to reduce N_2_O emissions.

## Materials and Methods

### Bacterial Strains, Growth Media, and Conditions

*Paracoccus denitrificans* was grown in a defined minimal medium which contained 29 mmol/L Na_2_HPO_4_, 11 mmol/L KH_2_PO_4_, 10 mmol/L NH_4_Cl, 0.4 mmol/L MgSO_4_, and supplemented with 30 mmol/L sodium succinate, 20 mmol/L NaNO_3_, and 2 mL/L Vishniac and Santer trace elements solution [130 mmol/L EDTA, 7.64 mmol/L ZnSO_4_, 25 mmol/L MnCl_2_, 18.5 mmol/L FeSO_4_,0.89 mmol/L (NH_4_)_6_Mo_7_O_24_, 6.4 mmol/L CuSO_4_,6.72 mmol/LCoCl_2_, 37.4 mmol/L CaCl_2_] (Crutzen et al., 2008).

For high N_2_O emitting culture conditions, CuSO_4_ was omitted from the trace elements solution as in Sullivan et al. (2013). Anaerobic batch cultures (200 mL) inoculated with 1% (v/v) of stationary phase cells that had been pre-grown in minimal media. Vessels used were 250 mL Duran bottles with screw-cap lids and gas-tight silicone septa. Cultures were sparged with N_2_ for 10 min to impose an anaerobic environment and incubated statically at 30°C. For zero N_2_O emitting conditions, aerobic conditions were created by using 50 ml of media in a 250 ml flask and shaking at 200 rpm at 30°C.

### Measurement of N_2_O Levels

Headspace gas samples (3 mL) were taken using a 5 mL gas-tight syringe (Hamilton) and stored in 3 mL pre-evacuated screw cap EXETAINER^®^ vials (Labco). N_2_O gas samples were analyzed by GC through injection of a 50 μL sample into a Clarus 500 gas chromatographer (PerkinElmer) with an electron capture detector and Elite-PLOT Q [DVB Plot column, 30 m × 0.53 mm ID, carrier: N_2_, make-up: 95% (v/V) argon/5% (v/v) methane]. Standards of N_2_O [5, 100, 1,000, 5,000, and 10,000 ppm (Scientific and Technical Gases)] were used to quantify N_2_O levels. Total N_2_O amounts were calculated by applying Henry’s Law constant for N_2_O at 30°C, KH cc of 0.5392. Values of N_2_O (in micromoles) were multiplied by two to adjust values to micromole N in the form of N_2_O (N⋅N_2_O), this takes into account of the stoichiometry of N in N_2_O.

### RNA Extraction

For RNA extraction, 30 mL of mid exponential phase cells (OD_600_ ≈ 0.4) was added to 12 mL of ice-cold 95% ethanol/5% phenol (pH = 4.3) (v/v) solution, and incubated on ice for 30 min to stabilize RNA and prevent degradation. RNA was isolated, using the Trizol method according to the protocol described in (Kröger et al., 2012). Trace DNA contamination was removed using Turbo DNA-free DNase (Ambion) and this was confirmed by PCR amplification of RNA samples using MyFi DNA polymerase (Bioline) according to the manufacturer’s instructions. RNA was quantified spectrophotometrically using a Nanodrop 2000 (Thermo Scientific), and integrity of RNA samples was analyzed using an Experion Automated Electrophoresis platform (Bio-Rad) using RNA StdSens chips (Bio-Rad) according to the manufacturer’s instructions.

### Library Preparation and Sequencing

Library preparation and sequencing were performed by Vertis Biotechnology AG, Germany. Briefly, the total RNA samples were split into two, one was enriched for the small RNA fractions < 200 nt (s) specifically using the RNeasy MinElute Cleanup Kit (Qiagen). Ribosomal RNA molecules were depleted from both samples using the Ribo-Zero rRNA Removal Kit for bacteria (Epicenter). The rRNA depleted RNA fractions were first treated with Tobacco Acid Pyrophosphatase (TAP, Epicenter). Afterward, oligonucleotide adapters were ligated to the 5′ and 3′ ends of the RNA samples. First-strand cDNA synthesis was performed using M-MLV reverse transcriptase and the 3′ adapter as primer. The resulting cDNAs were amplified by PCR using a high fidelity DNA polymerase. The cDNA was purified using the Agencourt AMPure XP kit (Beckman Coulter Genomics) and was analyzed by capillary electrophoresis. The cDNA pool was sequenced on an Illumina NextSeq 500 system using 75 bp read length.

### Identification and Analysis of sRNAs

Raw reads were trimmed then and aligned to the *P. denitrificans* genome (Genbank numbers: CP000489.1, CP000490.1 and CP000491.1). Bam files for each condition were converted to strand-specific wig files to allow viewing in IGB, alongside the annotated *P. denitrificans* genome. Both raw (fastaq files) and processed data (wig files) are available on the GEO database (Series record number: GSE85362)

Expression levels of each gene in the genome under each condition from the non sRNA enriched sample as RPKM = Reads Per Kb exon (contig) per Million mapped reads (Mortazavi et al., 2008) were also determined and so these can be directly compared to each other.

Candidate sRNAs were identified manually using wig files for the sRNA enriched fraction using the IGB browser as small (<200 bp) transcripts expressed from intergenic regions or antisense to characterized ORFs. In order to obtain normalized expression intensities of the read coverage depth for the sRNAs, the number of reads for the sRNA was normalized relative to the total number of reads in the library for each condition. Mfold was used to predict candidate sRNA secondary structure^1^ (Zuker, 2003). The nearest Rho-independent terminator to each sRNA was identified from the TransTerm^2^ (Kingsford et al., 2007). Potential gene targets for each sRNA were identified using TargetRNA2^3^ (Kery et al., 2014). A single biological replicate for each condition was used, as in the approach used by McClure et al. (2014).

### RT-PCR Validation of sRNAs

The method used was that described by Khoo et al. (2012). Briefly, purified RNA was reverse transcribed into cDNA with an oligo(dT)18 primer using RevertAid First Strand cDNA Synthesis Kit (Fermentas). cDNA was used as the template for PCR using MyFi polymerase together with primers that were designed based on the sequences of sRNA candidates (Supplementary Table S2). Amplified products were analyzed by 3% agarose gel electrophoresis with GeneRuler^TM^ Low Range DNA Ladder (ThermoFisher scientific) run in parallel. PCR products were purified with the QIAquick Gel Purification Kit (Qiagen, Germany) and confirmed by Sanger sequencing (Eurofins).

## Results

### Identification of 167 Putative sRNAs in *P. denitrificans*

*Paracoccus denitrificans* was grown to exponential phase (16 h, OD_600_ ≈ 0.4), under 3 different conditions, high N_2_O emitting anaerobic, low N_2_O emitting anaerobic and zero N_2_O emitting aerobic conditions. Different N_2_O conditions were established by growing *P. denitrificans* under different copper and oxygen regimes as described in Sullivan et al. (2013). This previous work showed that *P. denitrificans* grown anaerobically in a low copper media emitted approximately 1–2 mM N_2_O, whereas *P. denitrificans* grown anaerobically in an optimal copper media did not accumulate N_2_O. Therefore culturing *P. denitrificans* under these conditions provides a way to analyze the sRNA landscape of *P. denitrificans* under high and low N_2_O emitting conditions respectively. Furthermore, this earlier paper performed a transcriptomic analysis using a DNA microarray under the same high N_2_O emitting anaerobic and low N_2_O emitting anaerobic conditions. We were therefore able to subsequently compare our RNA-seq data to the gene expression changes previously reported. The additional condition of zero N_2_O emitting aerobically grown cultures allowed us to assess the effect of oxygen availability on sRNA expression in *P. denitrificans*.

To validate our culture conditions, N_2_O levels emitted from the different regimes were measured. The high N_2_O anaerobic culture produced 1.8 mM N_2_O and the low N_2_O anaerobic culture produced 0.05 mM N_2_O. This is in good agreement with the previous report by Sullivan et al. (2013) and provided a solid platform for RNA isolation from the 3 cultures. The RNA samples were split, one was enriched for sRNAs specifically and used for sRNA identification while the other was not enriched for sRNAs and was instead used to provide genome wide expression data. This resulted in roughly 20 million 75 bp reads for each culture condition. Under high N_2_O emitting anaerobic conditions, expression of *nosZ* was at 10 RKPM whereas under low N_2_O emitting anaerobic conditions expression of *nosZ* was at 120 RPKM. Therefore, expression of *nosZ* was approximately 12 fold lower than under high N_2_O emitting anaerobic conditions than under low N_2_O emitting anaerobic conditions. This was also consistent with the results as reported by Sullivan et al. (2013).

**FIGURE 1:**
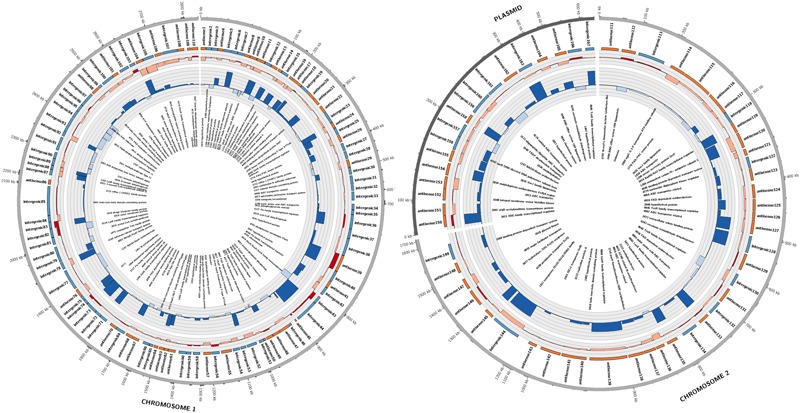
**Summary of sRNAs identified in the *P. denitrificans* transcriptome in high N_2_O (anaerobic), low N_2_O (anaerobic) and zero N_2_O emitting (aerobic) conditions.** Outer-to-inner rings: position in the *P. denitrificans* chromosome 1, chromosome 2 or plasmid; sRNA name; sRNA relative size and location, color-coded according to intergenic (blue) or antisense to ORF (orange) positions: sRNA expression level, color coded as increased expression in high N_2_O anaerobic compared to low N_2_O anaerobic (dark red), or lower expression in high N_2_O anaerobic compared to low N_2_O anaerobic (pale red), increased expression in low N_2_O anaerobic compared to zero N_2_O aerobic (dark blue) or lower expression in low N_2_O anaerobic compared to zero N_2_O aerobic (pale blue), with each ring representing increments of 2 log_2_-fold units of differential expression; predicted target for sRNA, Gene identifier (pden) number is included along with gene name when known. Note: for spacing purposes the gene names for predicted targets for 5 sRNAs on chromosome 1 could not be included, 4173 TonB-dependent receptor, 4861 ABC transporter related, 4986 ATP-NAD/AcoX, kinase 0810 solute-binding protein, 5071 hypothetical protein.

Candidate sRNAs were then conservatively identified from the reads obtained from the sRNA enriched condition manually using the IGB browser. A sRNA was called when a clearly enriched peak of <200 bp of at least 100 reads was expressed from an intergenic regions or antisense to characterized ORFs. Hundred and sixty seven sRNAs were identified across the whole genome as shown in **Figure 1**. Eighty four of these sRNAs were intergenic and 83 were antisense to ORFs. These were distributed across the entire genome with 110 on chromosome 1, 38 on chromosome 2 and 18 on the plasmid. A selection of 16 putative sRNAs (which were subsequently verified as being expressed) are shown in **Table 1**, with all the 167 sRNAs listed in Supplementary Table S1.

**Table 1:** The 16 sRNAs confirmed by RT-PCR as expressed in the *P. denitrificans* genome.

Name	Start site	End site	Size	Strand^A^	Reads in low N_2_O – o_2_^B^	Reads in high N_2_O – o_2_^C^	Reads in zero N_2_O + o_2_^D^	Putative target
Antisense 18	229605	229644	39	–	350	490	–	Pden_3255 Cupin 2, conserved barrel domain protein
Intergenic 39	744622	744742	120	+	377	274	335	Pden_3981 hypothetical protein
Intergenic 134	759993	760077	84	–	13968	17250	5830	Pden_5071 hypothetical protein
Antisense 146	1387901	1387989	88	+	363	652	82	Pden_4677 hydroxylase
Intergenic 36	726444	726596	152	+	4807	8771	472	Pden_5127 Fis family transcriptional regulator
Intergenic 60	1384880	1384960	80	+	175052	165043	120000	Pden_1824 hypothetical protein
Intergenic 100	2543197	2543334	137	+	1302	473	129	Pden_1294 hypothetical protein
Intergenic 12	87937	88007	70	+	170	138	942	Pden_0373 hypothetical protein
Antisense 13	90535	90607	72	+	133	142	–	Pden_1805 binding-protein-dependent transport system inner membrane protein
Intergenic 28	466884	466962	78	+	13822	14681	31052	Pden_2722 eﬄux-1 (HAE1) family protein
Antisense 29	497748	497832	84	+	130	57	346	Pden_1893 methionine-R-sulfoxide reductase
Antisense 115	273173	273337	164	+	562	292	903	Pden_2778 PAS/PAC sensor protein
Antisense 120	319497	319579	82	+	1209	189	17	Pden_1288 monovalent cation/H+ antiporter subunit C
Antisense 131	685081	685159	78	+	1922	2763	3999	Pden_4936 FAD dependent oxidoreductase
Intergenic 149	1574733	1574818	85	+	1204	1403	–	Pden_1089 binding-protein-dependent transport system inner membrane protein
Antisense 11	85367	85457	90	+	548	149	351	Pden_1370 hypothetical protein

*^A^Strand + positive DNA strand; - negative DNA strand; ^B^Reads in cells grown under low N_*2*_O anaerobic conditions normalized to total number of reads; ^C^Reads in cells grown under high N_*2*_O anaerobic conditions normalized to total number of reads; ^D^Reads in cells grown under zero N_*2*_O aerobic conditions normalized to total number of reads.*

### Confirmation of Expression of 16 sRNAs

**FIGURE 2:**
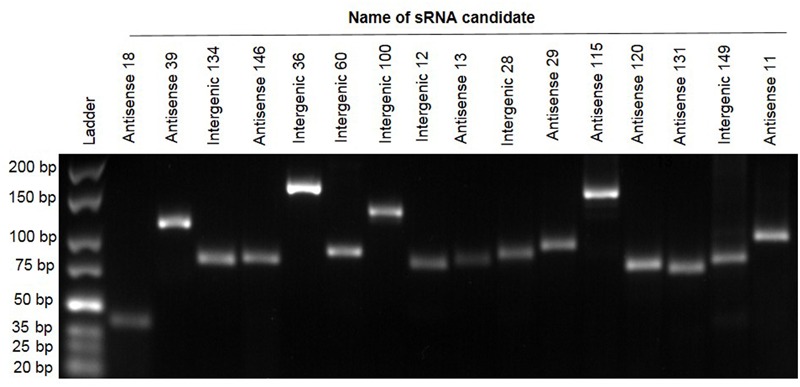
**Reverse transcriptase polymerase chain reaction validation of 16 sRNA candidates.** Electrophoresis of PCR products of 16 sRNAs on 3% agarose gel. Lane 1: GeneRuler Ultra Low Range DNA Ladder, Lanes 2–17. Confirmed sRNA candidates.

To independently confirm the presence and size of a selection of sRNAs predicted by RNA-seq we used RT-PCR (Pánek et al., 2008; Khoo et al., 2012; Panda et al., 2015; Kwenda et al., 2016). Briefly, RNA was reverse transcribed into cDNA. The cDNA produced was used as the template for PCR together with primers that were designed based on the relevant sequences of sRNA candidates (contained in Supplementary Table S2). Of 40 predicted sRNAs tested, 16 (40%) gave positive results of the expected size as shown in **Figure 2**. These PCR products were subsequently verified by Sanger sequencing. This proportion of successful validation of sRNAs (40%) fits well with other published data where validation is often successful around 40–50% of the time (examples include Kröger et al., 2012) where 60 new sRNAs were identified, of which 29 were confirmed (48%) and Khoo et al. (2012), where 15 were tested with 8 being verified (53%).

### sRNAs in *P. denitrificans* Are Highly Structured and Are Predicted to Have a Wide Range of Targets

**FIGURE 3:**
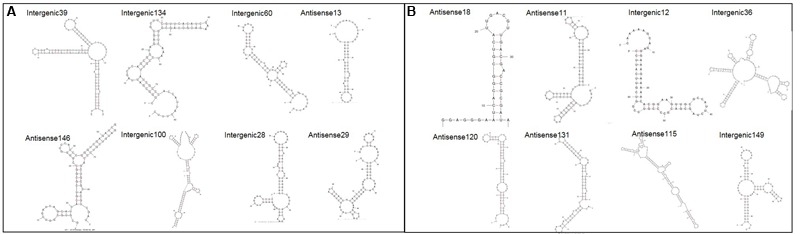
**Predicted secondary structures for 16 confirmed sRNAs.** The secondary structure for each confirmed sRNA was predicted using Mfold (Zuker, 2003). **(A)** Shows sRNAs which have putative homologues in other closely related bacteria and **(B)** shows sRNAs which have no putative homologues.

In order to gain more insight into the sRNAs identified various online tools were used to provide further information. Supplementary Table S1 provides all the information for all 167 sRNAs, but figures contained within this paper focus solely on the 16 confirmed sRNAs above for brevity. The nearest Rho-independent terminator to each sRNA was identified using TransTerm (Kingsford et al., 2007). The secondary structure of the sRNAs was predicted using Mfold with default parameters set (Zuker, 2003). All of the sRNAs were shown to have significant predicted secondary structure, with the predicted structures for the 16 confirmed sRNAs shown in **Figure 3**. Furthermore, 90% of the sRNAs (151/167 total) were predicted to form highly structured molecules including more than one hairpin loop. This is important as it shows that many of the sRNAs here have the potential to form complex conformations similar to those commonly associated with many other directly acting RNA transcripts, including known bacterial sRNAs.

Putative gene targets were predicted using TargetRNA (Kery et al., 2014) the three top targets (i.e., most energetically favorable) included in Supplementary Table S1 (with the top target highlighted in red). When we consider the top 3 targets for each sRNA, the most commonly predicted targets were transcriptional regulators such as the Xre, Fis and TetR families. These were predicted as targets in 118/167 sRNAs, the largest class by far. Another class of targets which was predicted in 100/167 of the sRNAs were transporters including metal and ABC transporters. There were also many cases where predicted targets included hypothetical proteins or proteins of unknown function (133/167 sRNAs). This information could potentially be helpful in eventually assigning a function to these unknown proteins.

### Conservation and Homolog Identification of *P. denitrificans* sRNAs

In order to see if any similar sRNAs had previously been identified, we searched the bacterial sRNA databases sRNATarBase (Cao et al., 2010) and BSRD (Li et al., 2013) using the BLAST options. We were initially surprised to see that when the 16 sRNAs from **Table 1** were input no sRNA homologues were identified in either database. However, these databases contain sRNAs identified from previous studies which have typically been performed in Gram-negative γ-proteobacteria such as *Escherichia coli* and *Salmonella*. On reflection, given the substantial difference in GC content between these organisms, it is perhaps not that surprising that no homologues to these putative *P. denitrificans* sRNAs were found. However it is possible that although the sequences of the sRNAs may have diverged significantly to prevent detection by sequence alignment alone, there may be structural conservation which would not be detected by this method.

In further analysis, the sequence conservation of novel sRNAs in other bacteria was investigated using BLASTn and the results shown in **Table 2**. A BLASTn comparison of each sequence was performed to all sequenced bacterial genomes (*E-*value = 10^-6^, word = 11). Only hits with nucleotide identity higher than 60% combined with coverage between query and subject sequence higher than 80% were considered to be conserved. Some 8/16 confirmed sRNAs (antisense 39, intergenic 134, intergenic 60, antisense 13, antisense 146, intergenic 100, intergenic 28 and antisense 29) were found to have conserved sequences in other α-proteobacteria, mainly in the *Rhodobacteraceae* family. It is expected that these conserved sRNAs may play a conserved role in such closely related species.

**Table 2:** Conservation of the sequences of sRNAs across different orders, classes, and species of proteobacteria.

sRNA	Class	Order	Species/Strains
Antisense 39	α-proteobacteria	*Rhodobacteraceae*	*Paracoccus aminophilus JCM 7686*
Intergenic 134	α-proteobacteria	*Rhodobacteraceae* *Rhizobiales*	*Paracoccus aminophilus JCM 7686* *Rhodobacter sphaeroides 2.4.1, Rhodobacter sphaeroides KD131, Rhodobacter sphaeroides ATCC 17029, Rhodobacter sphaeroides ATCC 17025, Rhodobacter capsulatus SB 1003* *Defluviimonas alba, Leisingera methylohalidivorans DSM 14336, Celeribacter marinus, Celeribacter indicus, Ruegeria sp. TM1040, Ruegeria pomeroyi DSS-3, Marinovum algicola DG 898, Phaeobacter gallaeciensis DSM 26640, Phaeobacter gallaeciensis 2.10, Phaeobacter inhibens DSM 17395, Jannaschia sp. CCS1, Rhodovulum sulfidophilum DSM 1374, Confluentimicrobium sp. EMB200-NS6*, *Ketogulonicigenium vulgare WSH-001, Ketogulonicigenium vulgare Y25, Roseibacterium elongatum DSM 19469, Mesorhizobium ciceri biovar biserrulae, Mesorhizobium huakuii, Mesorhizobium australicum WSM2073, Aminobacter aminovorans, Neorhizobium galegae Rhizobium leguminosarum*
	Terrabacteria group	*Chloroflexus Actinobacteria*	*Chloroflexus aurantiacus J-10-fl, Chloroflexus sp. Y-400-fl*, *Streptomyces sp. RTd22, Streptomyces sp. SAT1, Streptomyces iranensis, Streptomyces parvulus, Streptomyces ambofaciens, Streptomyces ambofaciens ATCC 23877, Streptomyces sp. S10(2016), Streptomyces albus J1074, Streptomyces reticuli, Streptomyces globisporus C-1027, Streptomyces sp. FR-008, Streptomyces hygroscopicus subsp. limoneus, Streptomyces hygroscopicus subsp. jinggangensis, Streptomyces sp. CFMR 7, Streptomyces pristinaespiralis, Streptomyces sp. PBH53 Streptomyces incarnatus, Streptomyces albulus, Streptomyces lydicus A02, Streptomyces nodosus, Streptomyces glaucescens, Streptomyces lividans TK24, Streptomyces davawensis JCM 4913, Micromonospora aurantiaca ATCC 27029, Thermobispora bispora DSM 43833, Amycolatopsis japonica, Modestobacter marinus, Blastococcus saxobsidens DD2.*
Intergenic 60	α-proteobacteria	*Rhodobacteraceae* *Sphingomonadales*	*Paracoccus aminophilus JCM 7686, Rhodobacter sphaeroides KD131, Rhodobacter sphaeroides ATCC 17025, Rhodobacter sphaeroides 2.4.1, Rhodobacter sphaeroides ATCC 17029, Rhodobacter capsulatus SB 1003, Celeribacter indicus, Celeribacter marinus, Marinovum algicola DG 898, Rhodovulum sulfidophilum DSM 1374, Defluviimonas alba, Roseobacter denitrificans OCh 114, Roseobacter litoralis Och 149, Roseibacterium elongatum DSM 19469, Ruegeria pomeroyi DSS-3, Ruegeria sp. TM1040, Confluentimicrobium sp. EMB200-NS6, Phaeobacter gallaeciensis DSM 26640, Phaeobacter gallaeciensis 2.10, Phaeobacter inhibens DSM 17395, Leisingera methylohalidivorans DSM 14336, Planktomarina temperata RCA23 Jannaschia sp. CCS1 Dinoroseobacter shibae DFL 12 = DSM 16493 Ketogulonicigenium vulgare, Ketogulonicigenium vulgare WSH-001, Ketogulonicigenium vulgare Y25, Sphingopyxis macrogoltabida, Sphingopyxis granuli, Novosphingobium sp. PP1Y, Novosphingobium pentaromativorans US6-1 Sphingomonas wittichii RW1, Sphingomonas sp. MM-1, Altererythrobacter atlanticus, Altererythrobacter marensis*
Antisense 13	α-proteobacteria	*Rhodobacteraceae* *Rhizobiales*	*Starkeya novella DSM 506, Bradyrhizobium sp. BTAi1, Xanthobacter autotrophicus Py2 Rhodopseudomonas palustris BisB18, Rhodopseudomonas palustris BisA53, Bradyrhizobium oligotrophicum S58, Jannaschia sp. CCS1, Bradyrhizobium sp. ORS278*
Antisense 146	α-proteobacteria	*Rhodobacteraceae Rhizobiales Rhodospirillaceae*	*Celeribacter indicus, Rhodobacter capsulatus SB 1003, Rhodobacter sphaeroides 2.4.1, Rhodobacter sphaeroides KD131, Rhodobacter sphaeroides ATCC 17029, Rhodobacter sphaeroides ATCC 17025, Pannonibacter phragmitetus, Hyphomicrobium nitrativorans NL23, Azospirillum lipoferum 4B, Steroidobacter denitrificans*
Intergenic 100	α-proteobacteria	*Rhodobacteraceae*	*Celeribacter indicus, Rhodobacter capsulatus SB 1003, Rhodobacter sphaeroides 2.4.1, Rhodobacter sphaeroides ATCC 17029.*
Intergenic 28	α-proteobacteria	*Rhodobacteraceae Rhizobiales*	*Paracoccus aminophilus JCM 7686, Rhodobacter sphaeroides 2.4.1, Rhodobacter sphaeroides KD131, Rhodobacter sphaeroides ATCC 17029, Rhodobacter sphaeroides ATCC 17025, Defluviimonas alba, Chelativorans sp. BNC1, Aminobacter aminovorans, Mesorhizobium ciceri biovar biserrulae, Mesorhizobium ciceri biovar biserrulae WSM1271, Mesorhizobium huakuii 7653R, Mesorhizobium australicum WSM2073, Mesorhizobium opportunistum WSM2075, Mesorhizobium loti MAFF303099, Aureimonas sp. AU20, Aureimonas frigidaquae*
	β-proteobacteria	*Alcaligenaceae Sulfuricellales Gallionellales*	*Bordetella pertussis 137, Bordetella pertussis B1920*, *Bordetella pertussis B1917, Bordetella pertussis 18323, Bordetella pertussis CS, Bordetella trematum, Bordetella bronchiseptica, Bordetella bronchiseptica 253, Bordetella bronchiseptica MO149, Bordetella hinzii, Bordetella petrii, Achromobacter xylosoxidans A8, Achromobacter xylosoxidans C54, Achromobacter xylosoxidans NH44784-1996, Achromobacter denitrificans, Castellaniella defragrans, 65Phen Pusillimonas sp. T7-7, Sulfuricella denitrificans skB26, Gallionella capsiferriformans ES-2*
Antisense 29	α-proteobacteria	*Rhodobacteraceae*	*Paracoccus aminophilus JCM 7686, Ruegeria sp. TM1040, Ruegeria pomeroyi DSS-3, Leisingera methylohalidivorans DSM 14336, Dinoroseobacter shibae DFL 12 = DSM 16493, Confluentimicrobium sp. EMB200-NS6, Rhodovulum sulfidophilum DSM 1374, Defluviimonas alba, Jannaschia sp. CCS1*

*BLASTn searches were performed (*E-*value = 10^-*6*^, word = 11) and classes and species where homology (query cover at least 80% and sequence identity at least 60%) to a given sRNA was observed are shown. Species which also encode *nosZ* are highlighted in red.*

The remaining eight confirmed sRNAs (intergenic 36, intergenic 12, antisense 29, antisense 11, antisense 115, antisense 120 and intergenic 149) showed no sequence homology to any other bacteria. Therefore, it seems likely that most of the sRNAs identified in our approach may be specific to closely related *Rhodobacterales* bacteria with some being species specific to *P. denitrificans*.

**FIGURE 4:**
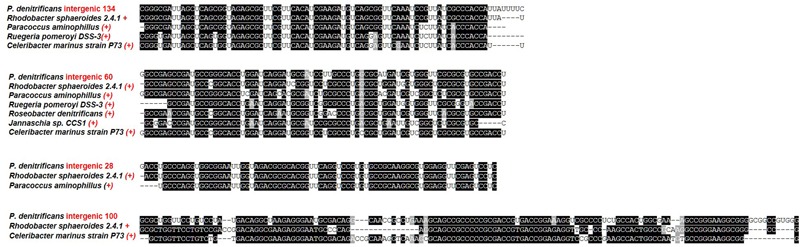
**Multiple sequence alignment of putative homologues to confirmed intergenic sRNAs in *P. denitrificans*.** +, indicates sRNA present in the same genetic context as in *P. denitrificans*, i.e., same gene up or downstream and (+) indicates sRNA present, but genetic context not conserved.

However, because the sequences of the sRNAs are conserved in other bacteria, this is not to say they are true sRNA candidates in other bacteria. In order to assign putative homologues to the 16 confirmed *P. denitrificans* sRNAs BLASTn searches of the sRNAs were performed as before, but in addition to this the genomic context of the sRNA was taken into account and the sequences aligned to the sRNA. The results are shown in **Figure 4**. Only intergenic sRNAs which had previously been confirmed as expressed were used for this analysis. For several sRNAs, intergenic 134, intergenic 60, intergenic 29 and intergenic 39, potential homologues with high sequence identity were found in genomes of bacterial species affiliated to the *Rhodobacterales.* Two of these sRNAs, intergenic 134 and intergenic 100, shared the same genetic context as well as significant sequence similarity with a putative sRNA in *R. sphaeroides*.

### Differential Expression of sRNAs under High and Low N_2_O Emitting Conditions

It was clear that many of the sRNAs were expressed at different levels under the experimental conditions used (Supplementary Table S1 includes expression values for all 167 sRNAs under all three conditions). For example, as shown in **Table 1**, sRNA intergenic 100 is expressed threefold higher under low N_2_O emitting conditions compared to under high N_2_O emitting conditions. Interestingly, analysis of the entire dataset showed 59/167 (35%) sRNAs were differentially expressed by twofold higher or lower (i.e., a ratio either < 0.5 or > 2 between high N_2_O emitting conditions and low N_2_O emitting conditions). Seven sRNAs showed a larger than fivefold change in expression between the two conditions (i.e., a ratio either < 0.2 or > 5 between conditions). A further 3 sRNAs showed a greater than 10-fold change in expression between the two conditions (i.e., a ratio either < 0.1 or > 10) between conditions). However, we acknowledge that future work is required to determine the significance of this differential expression.

Despite this, it is expected that the different levels of expression under different conditions will likely reflect the role of the sRNA in *P. denitrificans* physiology, with the 59 sRNAs showing increased expression under high N_2_O potentially playing a key role in the response to denitrification and N_2_O emissions.

### Differential Expression of sRNAs under Aerobic Zero N_2_O and Anaerobic Low N_2_O Conditions

From the conditions used it was also possible to compare the expression of sRNAs under aerobic zero N_2_O emitting conditions and anaerobic low N_2_O conditions. For example, as shown in **Table 1** sRNA intergenic 36 was expressed most highly under low N_2_O emitting anaerobic conditions, and showed an 18 fold reduction in expression under zero N_2_O emitting aerobic conditions. Analysis of all 167 sRNAs showed that 51/167 (31%) sRNAs were differentially expressed by 2 fold higher or lower (i.e., a ratio either < 0.5 or > 2 between low N_2_O emitting anaerobic conditions and zero N_2_O emitting aerobic conditions). Sixteen sRNAs showed a larger than fivefold change in expression between the two conditions (i.e., a ratio either < 0.2 or > 5 between conditions). A further 55 sRNAs showed a greater than 10-fold change in expression between the two conditions (i.e., a ratio either < 0.1 or > 10) between conditions). This analysis comes with the same caveat as before, that future work is required to determine the significance of this differential expression.

However, we do suggest it is likely that the different levels of expression under different conditions will likely reflect the role of the sRNA in *P. denitrificans*, with the 106 sRNAs showing increased expression under low N_2_O emitting anaerobic conditions potentially playing a key role in the response to anaerobic conditions specifically.

## Discussion

It has already been well established that various environmental factors including pH, aeration and metal availability affects production of N_2_O at an enzymatic level (Richardson et al., 2009). Despite this we lack detailed knowledge of the effects these factors can play at the transcriptional level. Recently, using microarrays, our laboratory demonstrated that copper availability affects transcription of the genes to produce enzymes required for N_2_O production in the model denitrifier *P. denitrificans*. This work provides a significant advance in understanding the N_2_O relevant transcriptional factors by identifying the sRNA landscape of *P. denitrificans* under high N_2_O and low N_2_O emitting conditions. Here we have shown that many sRNAs are expressed differentially under these conditions, suggesting a potential role for sRNAs in N_2_O production and/or reduction.

This work has revealed the expression of 16 sRNAs in *P. denitrificans*, which is the first description, to our knowledge, of sRNA expression in *P. denitrificans.* We foresee that the data provided in Supplementary Table S1, which contains information on all 167 sRNAs found will become a useful resource for the *P. denitrificans* and N-cycling community.

The putative target genes for many of our identified sRNAs included genes encoding products involved in transcriptional regulation such as the TetR family of regulators, which may act globally. This is consistent with other studies where global regulators are subject to regulation by multiple Hfq-dependent sRNAs in other species of bacteria (Lee and Gottesman, 2016). In addition to this many predicted targets of the sRNAs included proteins involved in transport. Interestingly this was the most commonly predicted target of sRNAs in the marine bacterium *Ruegeria pomeroyi* (Rivers et al., 2016). *R. pomeroyi* is closely related to *P. denitrificans* and it is possible that regulation of transporters may be a conserved role for sRNA across these related species. Also, many sRNAs identified here were predicted to interact with proteins of currently unknown function. A recent review compared predicted targets compared with true biological targets for organisms such as *E. coli* when many experimentally determined targets are known and concluded that there was a high number of true biological targets with relatively low scores from predictions (Pain et al., 2015). Nevertheless, it is useful to predict potential targets sRNAs as we have done here as this may help guide future research. However, it is clear that the only way to confirm a sRNA-target interaction is by experimental validation. Therefore, future work will concentrate on characterizing the functional roles and targets for putative sRNAs described here, particularly those we believe may function in the regulation of N_2_O production and/or reduction. It will also be interesting to see if any sRNAs are Hfq-dependent in the same way previously described sRNAs are in other bacterial species (Storz et al., 2011). *P. denitrificans* is predicted to encode an Hfq protein, which shows 95% sequence identity to *R. sphaeroides* Hfq (*e* value 5 × 10^-57^) and 54% sequence identity to *P. aeruginosa* Hfq (*e* value and 1 × 10^-28^) respectively (shown in Supplementary Figure S1). It has been shown that many sRNAs in these bacteria are indeed Hfq-dependent (Sonnleitner et al., 2006; Berghoff et al., 2011) so it seems likely that some *P. denitrificans* sRNAs could also be Hfq-dependent.

Half of the 16 confirmed sRNAs are conserved in other species in the α-proteobacteria in classes such as the *Rhodobacteraceae* and *Rhizobiales*. One of these eight sRNAs, intergenic 28, also had sequence homology to members of the β-proteobacteria in *Alcaligenaceae, Sulfuricellales* and *Gallionellales*. Interestingly, conserved hits in the *Alcaligenaceae* family included members of the *Bordetella* genus. These included various strains of the host restricted human pathogens *B. pertussis* and *B. bronchiseptica*, but also the environmental strain *B. petrii*. *B. petrii* has been isolated from various environmental niches such as river sediment and polluted soil and it has been suggested that it represents an evolutionary link between free-living environmental bacteria and the host-restricted obligate pathogenic *Bordetella* (Gross et al., 2008). It seems possible that this sRNA might therefore be an example of a genetic element found in soil dwelling bacteria such as *P. denitrificans* and *B. petrii* which is still retained in pathogens.

Also of note was sRNA intergenic 134 which has sequence homology to bacterial classes in the Terrabacteria group, *Chloroflexus* and *Actinobacteria.* This is intriguing as the terrabacteria are an evolutionary distinct clade to the hydrobacteria clade which include the α-proteobacteria. However, *Actinobacteria* are primarily soil dwelling organisms so it is possible that this sRNA plays an important role in the adaption to the soil environment of *Actinobacteria* and *P. denitrificans*.

The remaining 50% of the confirmed sRNAs have no sequence conservation to other related bacteria. This suggests that some *P. denitrificans* sRNAs are species specific while others are conserved in other closely related bacteria. Such an observation is consistent with the conservation seen between other species (Gottesman and Storz, 2011).

As these sRNAs were identified under high and low N_2_O emitting conditions respectively, we wanted to see if the sRNAs were present in bacterial species which had the ability to reduce N_2_O to N_2_ by encoding *nosZ.* The sequence of *P. denitrificans nosZ* was used as a query sequence for a BLASTn search (*E* value = 10^-6^, word = 11). Only hits with nucleotide identity higher than 60% combined with coverage between query and subject sequence higher than 80% were considered to be conserved and species which encoded *nosZ* are highlighted in **Table 2**. Seven out of the eight sRNAs have conserved sequences in several species which also encode *nosZ.* This may suggest that as the sRNA sequence and *nosZ* are found in the same species many times, *nosZ* expression could potentially be controlled by sRNAs.

Furthermore, this work focused on identifying classical intergenic and antisense to ORF sRNAs exclusively but there is increasing evidence that other classes of sRNAs exist in bacteria. sRNAs that exist intragenically or at 3′ and 5′ ends of ORFs have been validated (Vogel et al., 2003; Chao et al., 2012; Kröger et al., 2012). Further analysis of the data produced in this study will help show if this is the case in *P. denitrificans*, which could potentially increase the number of sRNAs in this organism significantly.

The long term goal of this work is to produce a compendium of transcriptional regulation information on denitrification in *P. denitrificans* as a model organism for this process. Better understanding of the intrinsic factors, such as sRNAs, which contribute to transcription of the N_2_O machinery will, in turn, help to inform strategies for mitigation of N_2_O emissions. More generally, this work, along with the work of many other laboratories in identifying a wide range of novel bacterial sRNAs, very much suggests that the prevalence and various roles of bacterial sRNAs are only just beginning to be appreciated.

## Author Contributions

HG designed experiments, acquired data, wrote, revised and approved final manuscript. LC acquired data, drafted manuscript and approved final manuscript. JM interpreted data, drafted manuscript and approved final manuscript. MA acquired data, drafted manuscript and approved final manuscript. AG designed experiments, drafted, revised and approved final manuscript. DR designed experiments, drafted, revised and approved final manuscript. GR designed experiments, wrote, drafted, revised and approved final manuscript.

## Conflict of Interest Statement

The authors declare that the research was conducted in the absence of any commercial or financial relationships that could be construed as a potential conflict of interest.

## Footnotes

1 http://unafold.rna.albany.edu/?q=mfold

2 http://transterm.cbcb.umd.edu/

3 http://tempest.wellesley.edu/btjaden/TargetRNA2/

## Abbreviations

BLAST: basic local alignment search tool
cDNA: complementary deoxyribonucleic acid
GC: gas chromatography
IGB: integrated genome browser
mRNA: messenger ribonucleic acid
N_2_O: nitrous oxide
RNA: ribonucleic acid
ORF: open reading frame
RT-PCR: reverse transcriptase polymerase chain reaction
sRNA: small ribonucleic acid

## References

[B1] Alloway B. J. (2008). *Micronutrient Deficiencies in Global Crop Production.* Dordrecht: Springer.

[B2] Berghoff B. A. Glaeser J. Sharma C. M. Zobawa M. Lottspeich F. Vogel J. (2011). Contribution of Hfq to photooxidative stress resistance and global regulation in *Rhodobacter sphaeroides*. *Mol. Microbiol.* 80 1479–1495. 10.1111/j.1365-2958.2011.07658 21535243

[B3] Cao Y. Wu J. Liu Q. Zhao Y. Ying X. Cha L. (2010). sRNATarBase: A comprehensive database of bacterial sRNA targets verified by experiments. *RNA* 16 2051–2057. 10.1261/rna.2193110 20843985 PMC2957045

[B4] Chao Y. Papenfort K. Reinhardt R. Sharma C. M. Vogel J. (2012). An atlas of Hfq-bound transcripts reveals 3’ UTRs as a genomic reservoir of regulatory small RNAs. *EMBO J.* 3120 4005–4019. 10.1038/emboj.2012.229 PMC3474919 22922465

[B5] Crutzen P. J. Mosier A. R. Smith K. A. Winiwarter W. (2008). N_2_O release from agro-biofuel production negates global warming reduction by replacing fossil fuels Atmos. *Chem. Phys.* 8 389–395.

[B6] Gottesman S. Storz G. (2011). Bacterial small RNA regulators: versatile roles and rapidly evolving variations. *Cold Spring Harb. Perspect. Biol.* 3:12 10.1101/cshperspect.a003798 PMC3225950 20980440

[B7] Gross R. Guzman C. A. Sebaihia M. dos Santos V. A. Pieper D. H. Koebnik R. (2008). The missing link: *Bordetella petrii* is endowed with both the metabolic versatility of environmental bacteria and virulence traits of pathogenic *Bordetella*. *BMC Genomics* 9:449 10.1186/1471-2164-9-449 PMC2572626 18826580

[B8] Kery M. B. F. M. Livny J. Tjaden B. (2014). TargetRNA2: identifying targets of small regulatory RNAs in bacteria. *Nucleic Acids Res.* 42 124–129. 10.1093/nar/gku317 PMC4086111 24753424

[B9] Khoo J. S. Chai S. F. Mohamed R. Nathan S. Firdaus-Raih M. (2012). Computational discovery and RT-PCR validation of novel *Burkholderia* conserved and *Burkholderia pseudomallei* unique sRNAs. *BMC Genomics* 13(Suppl. 7):S13 10.1186/1471-2164-13-S7-S13 PMC3521395 23282220

[B10] Kingsford C. L. Ayanbule K. Salzberg S. L. (2007). Rapid, accurate, computational discovery of Rho-independent transcription terminators illuminates their relationship to DNA uptake. *Genome Biol.* 8:R22 10.1186/gb-2007-8-2-r22 PMC1852404 17313685

[B11] Kröger C. Dillon S. C. Cameron A. D. Papenfort K. Sivasankaran S. K. Hokamp K. (2012). The transcriptional landscape and small RNAs of *Salmonella enterica* serovar Typhimurium. *Proc. Natl. Acad. Sci. U.S.A.* 109 1277–1286. 10.1073/pnas.1201061109 PMC3356629 22538806

[B12] Kwenda S. Gorshkov V. Ramesh A. M. Naidoo S. Rubagotti E. Birch P. R. (2016). Discovery and profiling of small RNAs responsive to stress conditions in the plant pathogen *Pectobacterium atrosepticum*. *BMC Genomics* 12:47 10.1186/s12864-016-2376-0 PMC4710047 26753530

[B13] Lee H. J. Gottesman S. (2016). sRNA roles in regulating transcriptional regulators: Lrp and SoxS regulation by sRNAs. *Nucleic Acids Res.* 44 6907–6923. 10.1093/nar/gkw358 27137887 PMC5001588

[B14] Li L. Huang D. Cheung M. K. Nong W. Huang Q. Kwan H. S. (2013). BSRD: a repository for bacterial small regulatory RNA. *Nucleic Acids Res.* 41 233–238. 10.1093/nar/gks1264 PMC3531160 23203879

[B15] McClure R. Tjaden B. Genco C. (2014). Identification of sRNAs expressed by the human pathogen *Neisseria gonorrhoeae* under disparate growth conditions. *Front. Microbiol.* 2014:5456 10.3389/fmicb.2014.00456 PMC4148029 25221548

[B16] Moll I. Leitsch D. Steinhauser T. Bläsi U. (2003). RNA chaperone activity of the Sm-like Hfq protein. *EMBO Rep.* 4 284–289. 10.1038/sj.embor.embor772 12634847 PMC1315900

[B17] Mortazavi A. Williams B. A. McCue K. Schaeffer L. Wold B. (2008). Mapping and quantifying mammalian transcriptomes by RNA-Seq. *Nat. Methods* 5 621–628. 10.1038/nmeth.1226 18516045 PMC13303166

[B18] Pain A. Ott A. Amine H. Rochat T. Bouloc P. Gautheret D. (2015). An assessment of bacterial small RNA target prediction programs. *RNA Biol.* 12 509–513. 10.1080/15476286.2015 25760244 PMC4615726

[B19] Panda G. Tanwer P. Ansari S. Khare D. Bhatnagar R. (2015). Regulation and RNA-binding properties of Hfq-like RNA chaperones in *Bacillus anthracis*. *Biochim. Biophys. Acta* 1850 1661–1668. 10.1016/j.bbagen.2015.03.016 25863287

[B20] Pánek J. Bobek J. Mikulík K. Basler M. Vohradský J. (2008). Biocomputational prediction of small non-coding RNAs in *Streptomyces*. *BMC Genomics* 9:217 10.1186/1471-2164-9-217 PMC2422843 18477385

[B21] Pomowski A. Zumft W. G. Kroneck P. M. Einsle O. (2011). N_2_O binding at a [4Cu:2S] copper-sulphur cluster in nitrous oxide reductase. *Nature* 477 234–237. 10.1038/nature10332 21841804

[B22] Ravishankara A. R. Daniel J. S. Portmann R. W. (2009). Nitrous Oxide (N_2_O): the dominant ozone-depleting substance emitted in the 21st century. *Science* 326 123–125. 10.1126/science.1176985 19713491

[B23] Richardson D. Felgate H. Watmough N. Thomson A. Baggs E. (2009). Mitigating release of the potent greenhouse gas N_2_O from the nitrogen cycle - could enzymic regulation hold the key? *Trends Biotechnol.* 27 388–397. 10.1016/j.tibtech.2009.03.009 19497629

[B24] Rivers A. R. Burns A. S. Chan L. K. Moran M. A. (2016). Experimental identification of small non-coding RNAs in the model marine bacterium *Ruegeria pomeroyi* DSS-3. *Front. Microbiol.* 29:380 10.3389/fmicb.2016.00380 PMC4809877 27065955

[B25] Sonnleitner E. Schuster M. Sorger-Domenigg T. Greenberg E. P. Bläsi U. (2006). Hfq-dependent alterations of the transcriptome profile and effects on quorum sensing in *Pseudomonas aeruginosa*. *Mol. Microbiol.* 59 1542–1558. 10.1111/j.1365-2958.2006.05032.x 16468994

[B26] Storz G. Vogel J. Wassarman K. M. (2011). Regulation by small RNAs in bacteria: expanding frontiers. *Mol. Cell.* 43 880–891. 10.1016/j.molcel.2011.08.022 21925377 PMC3176440

[B27] Sullivan M. J. Gates A. J. Appia-Ayme C. Rowley G. Richardson D. J. (2013). Copper control of bacterial nitrous oxide emission and its impact on vitamin B12-dependent metabolism. *Proc. Natl. Acad. Sci. U.S.A.* 110 19926–19931. 10.1073/pnas.1314529110 24248380 PMC3856849

[B28] Vogel J. Bartels V. Tang T. H. Churakov G. Slagter-Jäger J. G. Hüttenhofer A. (2003). RNomics in *Escherichia coli* detects new sRNA species and indicates parallel transcriptional output in bacteria. *Nucleic Acids Res.* 31 6435–6443. 10.1093/nar/gkg867 14602901 PMC275561

[B29] Zuker M. (2003). Mfold web server for nucleic acid folding and hybridization prediction. *Nucleic Acids Res.* 31 3406–3415. 10.1093/nar/gkg595 12824337 PMC169194

[B30] Zumft W. G. Kroneck P. M. (2006). Respiratory transformation of nitrous oxide (N_2_O) to dinitrogen by bacteria and archaea. *Adv. Microb. Physiol.* 52 100–227. 10.1016/S0065-2911(06)52003-X 17027372

