# Supplementary material for: Genome-Wide Discovery of Putative sRNAs in Paracoccus denitrificans Expressed under Nitrous Oxide Emitting Conditions
Source: Front Microbiol. 2016 Nov 14;7:1806. doi: 10.3389/fmicb.2016.01806 (PMC5107571; doi:10.3389/fmicb.2016.01806)
Supplement: Supplementary file 2 [file Table_2.docx]

**Supplementary Table 2:** **Nucleotide sequences of PCR primers for amplifying sRNA genes**

| Name of sRNA | Forward primer (5’-3’) | Reverse primer (5’-3’) | sRNA confirmed? |
| --- | --- | --- | --- |
| Antisense 18 | GGAGGGAATCACGGCG | TATCGCGGTCGTCAA | 🗸 |
| Antisense 24 | TAGGTTAAATGGCCGGA | TAGGTTAAATGGCCGGA |  |
| Antisense 39 | CCGGGCCTGCTCGTGCATGT | ACCGCCAGCGTCGAACCGTA | 🗸 |
| Intergenic 71 | AGGCAAGGCGAGGGGCGTGC | GCGTCCATCGCCGCCTGCCC |  |
| Antisense 8 | CACAAGAGCGGCCCAGCA | AAAGTAACAAAGGCCACCA |  |
| Antisense 119 | CATAACCTGAAGGTCGTAGG | GCGCAAGTAAAGATGGTTGC |  |
| Intergenic 134 | CGGGCGATTAGCTCAGCGG | GAAAATAATGGTGGGC | 🗸 |
| Antisense 146 | GCGTGCGGTCGATCAGCATC | ATCGACGTGCGCAGCTGGGA | 🗸 |
| Intergenic  100 | GCGCTGGTTCCTGTCCTATGAC | CCCACGGCCGCCCCGCCTTCCCGGCTTT | 🗸 |
| Intergenic 161 | ATTTTATACAATTCAT | CGGGCTATACCCGGGT |  |
| Intergenic 3 | GTGCCGGAAAGCAA | AAGATGATGGCGCGC |  |
| Intergenic 31 | TTCTCGCTCGTCATC | CAAAATACTCAGGA |  |
| Intergenic 36 | GCGAGTGTGTGATTCG | AAGGTCCAGATGTTT | 🗸 |
| Intergenic 37 | CAGGAAAGGCCC | CCTTTCAGCATAT |  |
| Intergenic 66 | CTGGCGGGTTTCTGC | TCACCGAGGGGG |  |
| Intergenic 60 | GGCCGAGCCGATG | AGGTCGGCACG | 🗸 |
| Intergenic 88 | TCAGTTAATCAGT | AAAAAGCCGGACCCG |  |
| Intergenic 23 | AGCGCCTCACGGCTGA | AAGGATCGGCCCCG |  |
| Antisense 137 | GTCGTCGATCTCTC | AGGAGCTATCAGAG |  |
| Antisense 162 | ATGGCGCATGTCGCA | GGTGTAGCGCGA |  |
| Antisense 1 | GCGAGATAGGCTTCGA | TCAACGACCGCTC |  |
| Intergenic 2 | AATACTTTGGTCC | CGGGTCCGGCCGC |  |
| Antisense 11 | GGATCTTGCCT | GCCGGCGAACCT | 🗸 |
| Intergenic 12 | GTAGATGGGGCAGAT | CGGCGGTTATGTT | 🗸 |
| Antisense 13 | ACTCTGGAGCCTG | GTGGATCTGAC | 🗸 |
| Antisense 16 | CTGGCGAAGCCA | ATACAGCCGGGC |  |
| Antisense 20 | GCCGAGTGCCT | GCCGCCGTCGATG |  |
| Antisense 26 | CCTGCCTCGATCAGC | CGACGTGGACGG |  |
| Intergenic 28 | GACCTGCCCAGGT | GAGGACTCGAACC | 🗸 |
| Antisense 29 | GGCCGGTAAAGAC | AGGTGATGGTCCAG | 🗸 |
| Antisense 112 | TTGAAGCGGGCCGGGGTC | ACGCCTGGGCCTCGGACC |  |
| Antisense 115 | CTTTGGCGAAACGGTCCT | AGCACGACCGCGACCCGGA | 🗸 |
| Antisense 120 | TCATCCGAGCGGGATCTCC | CTCGGGTCCAGAGCGTCGT | 🗸 |
| Antisense 123 | AGCGCGGGCGCCGCCGCT | CCCAGGCCGACGGCT |  |
| Antisense 137 | GTCGTCGATCTCTCGCC | GCTCAGGAGCTATCAG |  |
| Antisense 131 | ATCACCCGGCTGTCGA | CGTGCGCCACAACTCGA | 🗸 |
| Antisense 140 | TCCGCCGGGGAGACCCATG | AAGTGGTGGGCGACCCT |  |
| Antisense 147 | TCCTCGCGGCCCATCGGGTTG | CGCCGGCACCGCGCGCGAGGA |  |
| Intergenic 149 | ACGGTTCCGGCGCGATCTG | GGGCAGCCCCGGCCTTGCCC | 🗸 |
