# Supplementary figures and images for: Genome-Wide Discovery of Putative sRNAs in Paracoccus denitrificans Expressed under Nitrous Oxide Emitting Conditions
Source: Front Microbiol. 2016 Nov 14;7:1806. doi: 10.3389/fmicb.2016.01806 (PMC5107571; doi:10.3389/fmicb.2016.01806)

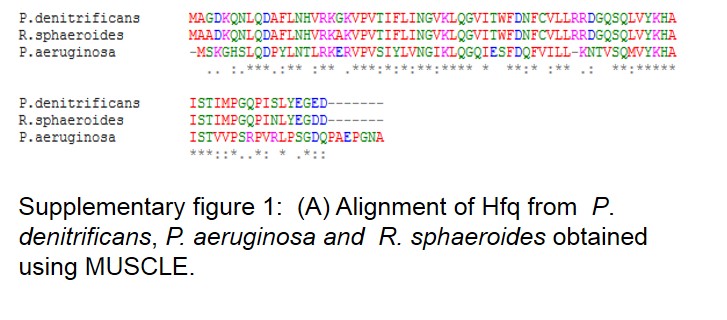

Supplement: Supplementary file 3 [file Image_1.JPEG]
